# Psychoanalytic Theories Do Not Support a Unidimensional View of Personality Functioning

**DOI:** 10.1002/pmh.70095

**Published:** 2026-08-12

**Authors:** Orestis Zavlis

**Affiliations:** ^1^ Unit of Psychoanalysis University College London London UK

## Abstract

Contemporary researchers tend to invoke psychoanalytic theories to support the view of personality functioning as a “unitary capacity” involving “psychological maturity.” In this piece, I argue against this false equivalence by raising three points. First, I show that psychoanalysis is not a homogenous discipline that converged on a shared developmental ontology, but rather a deeply heterogenous tradition marked, if anything, by persistent disagreement. Second, I briefly review commonly‐cited psychoanalytic theories (including classic Freudian, ego psychological, and object relational theories) to illustrate how none of them supports the view of personality functioning as a “unitary capacity.” Third, I argue that the origins of the construct “personality functioning” are rooted more in descriptive empirical findings rather than traditional psychoanalytic perspectives. Finally, I conclude by suggesting that researchers should stop flattening psychoanalytic theory to support their “unitary” perspective and should instead conduct rigorous empirical tests to determine whether there is any merit in their otherwise theoretically empty viewpoint.

I read with interest the commentary by Hopwood (2026) on the distinction among four ways of conceptualizing personality functioning. Although I found most of Hopwood's (2026) descriptions accurate, one of them (viz., the “psychodynamic”) appeared to me as largely misrepresented because it was falsely equated with a “unidimensional” view of human development. In the spirit of theoretical fidelity, I briefly outline below three key reasons why psychoanalytic theories cannot be invoked to support the view of personality functioning as a unidimensional entity speaking to “psychological maturity” (for a thorough discussion of the same topic, see Zavlis 2026d).

To begin with, it is first worth clarifying that psychoanalysis is not a monolithic discipline that converged on a shared developmental ontology, but rather a deeply heterogeneous tradition typified, if anything, by persistent disagreement (see Mitchell and Black 2016). To briefly illustrate some of these disagreements, ego psychologists (like Anna Freud and Heinz Hartmann) have long held that psychological development unfolds along a specific line of *self‐regulation* or *ego strength* (like the management of anxiety, impulse, and conflict); the object relations theorists (like Melanie Klein and Wilfred Bion) rejected this self‐focused emphasis and instead suggested that development lies in the *internalization of early relationships*; and finally, the radical post‐structuralists (like Lacan and Laplanche) parted away from mainstream psychoanalysis altogether by denying the very existence of a *coherent personality and sense of self* (in both mental health and illness). Interestingly, this heterogeneity of psychoanalysis is itself telling: Because psychological development could not be coherently reduced to “one process,” many theories naturally emerged to focus, at different times across history, on particular aspects of human development (like the development of self, affect regulation, mentalization, “good enough” ways of relating, ways of defending, ways of attaching, and ways of desiring; see Fonagy and Target 2003). In that sense, if anything characterizes psychoanalysis, it is the exact opposite of a single axis of human development; instead, it is an emphasis on multiple, interacting lines of human development (see Galatzer‐Levy 2017).^1^

Second, beyond psychoanalysis as a general discipline, it is worth noting more specifically that none of the psychoanalytic theories cited by Hopwood (2026) or related authors (e.g., Gilbert 2026; Hopwood 2025) ever promoted a singular view of human development or human personality. Indeed, if anything, each of these theories focused on the development of *specific psychological capacities*. For instance, Freud's (1905/1953) psychosexual stages focused not on a general capacity for “maturity,” but on the development of *sexuality*: specifically, about how scattered infantile drives (organized around different erotogenic zones, like the oral, anal, and genital) gradually develop into a single sexual organization that could lead on to different character traits later in life (e.g., being fixated in the oral stage leading to dependent traits while being fixated in the anal stage obsessive traits). Likewise, Erikson's (1950/1963) psychosocial model described a sequence of eight qualitatively distinct crises, each centered on a *specific psychological domain* (e.g., trust vs. mistrust; autonomy vs. shame; etc.), implying that mental illness was typified by specific failures in specific domains (e.g., a failure of basic trust leading to schizoid withdrawal, and a failure of autonomy leading to compulsive ways of being), not as a general impairment of “maturity” that cuts across all of these domains. Moreover, Klein's (1946/1975) object relations theory focused not on general aspects of “personality” but on *specific ways of relating* (viz., the paranoid‐schizoid vs. depressive positions), with mental health being defined as the flexible movement between them and illness as a rigid fixation in one of them. Finally, Kernberg never suggested that personality reflects a single capacity of “maturity”; instead, Kernberg suggested that personality can be distilled in *three specific kinds of* “personality‐related” *difficulties* (viz., the neurotic, the borderline, and the psychotic kinds of difficulties), which can be organized, to use Kernberg's (1970, 802–803) own terms, “for convenience” (i.e., descriptively rather than substantively) along a continuum of severity (see Zavlis 2026b for a detailed outline on why this dimension of severity is descriptive rather than substantive).^2^ As we can see, then, none of these (and related; see Zavlis 2026d) psychoanalytic theories support the view of personality functioning as a one‐dimensional axis of “developmental maturation” (Hopwood 2026, 3).^1^

Finally, the last point worth noting is that while psychoanalysis has been retroactively invoked to support the view of personality functioning as a “unitary entity” (e.g., Gilbert 2026; Hopwood 2025, 2026), this is not at all what the history of this construct shows when examined closely. Indeed, as I have explained at greater length elsewhere (Zavlis 2026d), the construct “personality functioning” did not emerge as the logical endpoint of psychoanalysis but rather as the empirical product of an attempt made by Bender et al. (2011, 332) to develop “a scale of severity of impairment in personality functioning.” Although this scale was informed by psychoanalytic content, the overall task was motivated by a well‐known descriptive finding, namely, the positive correlations of various personality disorder indicators (see Bender et al. 2011, 332). Moreover, the overall outcome of this task was not a *mechanistic theory* of how personality develops over time but rather a *descriptive index of severity*, summarizing what the various indicators of personality disorder have in common. In the face of this historical arc, then, it is misleading to suggest that it was psychodynamic theory that motivated the unitary structure of “personality functioning”; instead, the historically accurate reading is that a descriptive data pattern motivated researchers to create a putatively “unidimensional” questionnaire, and only after they have created it to flatten psychodynamic theory in order to retroactively justify it—all while neglecting the fact that no psychodynamic theory was ever unidimensional to begin with!

To conclude, then, there is no evidence to suggest that psychoanalysis ever endorsed the view of personality (functioning) as a single capacity relating to “maturity,” and the persistent appeal to psychoanalytic authority to ground such a viewpoint is, in my view, both historically inaccurate and theoretically counterproductive. Indeed, if anything, this appeal does more harm than good to Hopwood's (2026, 4) own case because, as he himself explains, psychoanalysis has unfavorable connotations, meaning that there might be little, if any, rhetorical gain from appeals to it. Were he to advance his thesis on contemporary five‐factor evidence instead, where a more circumscribed “maturity principle” has accrued some empirical support (e.g., the age‐graded tendency for traits such as conscientiousness and emotional stability to increase in their average levels across adult life; Bleidorn et al. 2022), the argument would have been more accurate and convincing. Yet even such an argument would still be incomplete because it does not speak about the development of “all personality” (e.g., all traits, or all adaptations, or all mechanisms of personality), leaving perhaps little‐to‐no probability for the possibility of a unidimensional view of personality (e.g., DeYoung et al. 2026; Zavlis 2026a) or its pathology (e.g., Zavlis 2026c).

## Funding

The author received no specific funding for this study.

## Data Availability

Data sharing not applicable to this article as no datasets were generated or analysed during the current study.

## Endnotes

^1^
At this point, I can anticipate that Hopwood (2026) and related theorists (e.g., Gilbert 2026), will counter‐claim that I am somehow conflating “general features of personality” (like the general factor of personality pathology) with “specific features of personality” (like specific, stylistic traits). However, this argument fails from within the tradition of psychoanalysis itself because psychoanalysis never bifurcated personality in this way. Instead, and consistent with my argument in this paper, psychoanalytic theories dealt with specific lines of development (like the development of self, ego, identity, and ways of relating). Moreover, to the extent that psychoanalysis ever viewed these lines of development in a “unitary” manner, this is also irrelevant to Hopwood's argument because once again these lines of development are specific processes (that speak about specific, unitary capacities), not general processes (that would reduce all such specific, unitary capacities to one general, unitary capacity). In this way, psychoanalysis can be interpreted as being entirely antithetical to the view that psychological development could ever be “one thing” precisely because it focused on the view that development is “many different things.”

^2^
It is important to emphasize this point once more because Kernberg is the most cited psychoanalyst in support of the view of “personality functioning” as “unitary substantive entity.” His own writings, however, tell a radically different story. Specifically, Kernberg's (1970, 1975) writings suggest that the general dimension of personality severity is not a substantive developmental process, but rather a descriptive (and nonlinear) continuum that is used “for convenience” so as to organize three types of “personality organization” problems, namely, the neurotic, the borderline, and the psychotic problems (Kernberg 1970, 802–803). In that sense, for Kernberg, the way personality is “organized” is something that is relevant for understanding *descriptively* the severity of major forms of psychopathology (neurosis, borderline, and psychosis), but not something that can be used *substantively* to explain the developmental origins of any of these psychopathologies. Again, for a detailed review on this topic, see Zavlis (2026b).
